# Automated phenotyping of ophthalmologic diseases from routine medical records using small language models and the human phenotype ontology (HPO)

**DOI:** 10.1038/s41598-026-51512-z

**Published:** 2026-05-09

**Authors:** Binh Duong Thai, Sebastian Arens, Thomas Reinhard, Daniel Böhringer

**Affiliations:** Medical Faculty, Eye Center, University Medical Center Freiburg, Freiburg, Germany and Albert Ludwigs University Freiburg, Killianstraße 5, 79106 Freiburg, Germany

**Keywords:** Artificial intelligence, Human phenotype ontology, Ophthalmological registries, Validation study, Computational biology and bioinformatics, Diseases, Health care, Medical research

## Abstract

Automated phenotyping in ophthalmology requires accurate standardization of clinical terms to facilitate interoperability and research. This study evaluates the suitability of the human phenotype ontology (HPO) for automated extraction of ophthalmic phenotypes from narrative documentation. We developed a locally operated AI pipeline combining text segmentation and negation detection based on a small language model (PHI-4) with a dense retrieval approach using an augmented multilingual HPO catalog. Synonyms were incorporated into the HPO during training on anonymized consecutive physician letters. To validate the pipeline, 175 anterior segment and fundus descriptions from randomly picked medical records were manually annotated with HPO terms as ground truth. Overall, 342 HPO terms were identified manually (on average 2.53 terms per document), with 341 retrieved by the pipeline (on average 2.52 terms per document). Performance metrics showed a median Jaccard similarity of 0.67, precision of 0.83, recall of 0.82, and F1 score of 0.80. These results demonstrate that our AI pipeline effectively extracts standardized HPO terms from free-text ophthalmic findings. Integration of this pipeline into clinical information systems may enhance data interoperability and reduce manual coding workload in ophthalmology practices in the future.

**Supplementary Information:**

The online version contains supplementary material available at 10.1038/s41598-026-51512-z.

## Introduction

Routine clinical documentation, particularly in high-volume specialties such as ophthalmology, contains vast amounts of detailed and clinically relevant information that remain largely untapped^1^. Physicians’ letters often include nuanced descriptions of ophthalmic phenotypes essential for both clinical care and research^1^. However, the efficient utilization of this information is hindered by varied terminology, inconsistent manual annotation, and the resulting lack of interoperability across healthcare sites. To overcome these limitations and enable automated medical registries, a paradigm shift toward automated extraction and standardization of clinical data is required, supported by the development of a robust and versatile vocabulary code.

The human phenotype ontology (HPO) has gained global acceptance as a structured nomenclature for phenotypic abnormalities, providing a standard for rare disease diagnosis, patient similarity analysis, and translational research^2^. The HPO has a widespread application in the domains of clinical diagnostics and translational research. These include, but are not limited to, the following: genomic diagnostics, gene-disease discovery, and cohort analysis^2^. Large language models demonstrate substantial performance for automated HPO code extraction, with retrieval augmented generation approaches achieving precision of 0.81–0.84, recall of 0.76–0.78, and F1 scores of 0.78–0.80, significantly outperforming traditional concept-recognition tools like Doc2HPO and ClinPhen (*p* < 0.00001)^3^.

In medicine, multimodal data are widespread and essential for supporting informed clinical decision-making^4^. These data encompass a wide range of modalities, including medical imaging such as MRI and CT, time-series data from wearable sensors and electronic health records, audio recordings such as cardiac and respiratory sounds and patient interviews, textual sources such as clinical notes and research articles, video data such as surgical procedures, and omics data such as genomics and proteomics^4^. With the rise of AI-driven techniques recently, particularly those employing large language models (LLMs) and deep retrieval strategies, AI is transforming the field of information extraction in medicine, offering superior results in processing and annotating clinical texts across various specialties^4,5^. Research studies suggest an increasing utility in automated detection of clinical findings, disease classification, and the execution of chart review tasks^6^. Large language models such as GPT-4 could achieve comparable or superior performance in related clinical phenotyping tasks, with note-level F1 scores of at least 0.90 for disease behavior extraction and 98.1% accuracy for oncologic phenotype identification^7,8^. When these AI systems are integrated with structured vocabulary such as HPO, they could potentially present significant advantages, including automatic deep phenotyping, higher information richness, and support for research and registry tasks that demand phenotypic precision.

The present study evaluates a general data protection regulation (GDPR) compliant locally deployed artificial intelligence (AI) pipeline that integrates text segmentation, negation recognition based on a small language model, and dense retrieval utilizing an expanded multilingual HPO thesaurus. The performance of this pipeline is then assessed and validated by comparing its automated extraction results with manual, expert annotations (ground truth) from anonymized clinical letters.

## Methods

### AI pipeline architecture

The computational framework of the pipeline integrates two deep learning models: the Phi-4 language model and the multilingual-e5-instruct embedding model.

Phi-4 (Microsoft) is a 14-billion parameter model optimized for advanced reasoning and instruction adherence^9^. It was selected for its balance between computational efficiency and high-fidelity text processing, making it suitable for deployment in resource-constrained environments. The model’s training involved a mix of synthetic datasets and filtered academic corpora, refined via supervised fine-tuning and direct preference optimization^10^.

For dense retrieval, we utilized the multilingual-e5-instruct model (Microsoft). Based on the XLM-RoBERTa-large architecture, this model generates 1024-dimensional dense vector representations. It is optimized for semantic search and retrieval across over 100 languages, having been fine-tuned on a massive corpus of text pairs and synthetic datasets^11,12^. This architecture allows for robust cross-lingual understanding and precise semantic matching within complex clinical data.

### Procedural framework

**Fig. 1:**
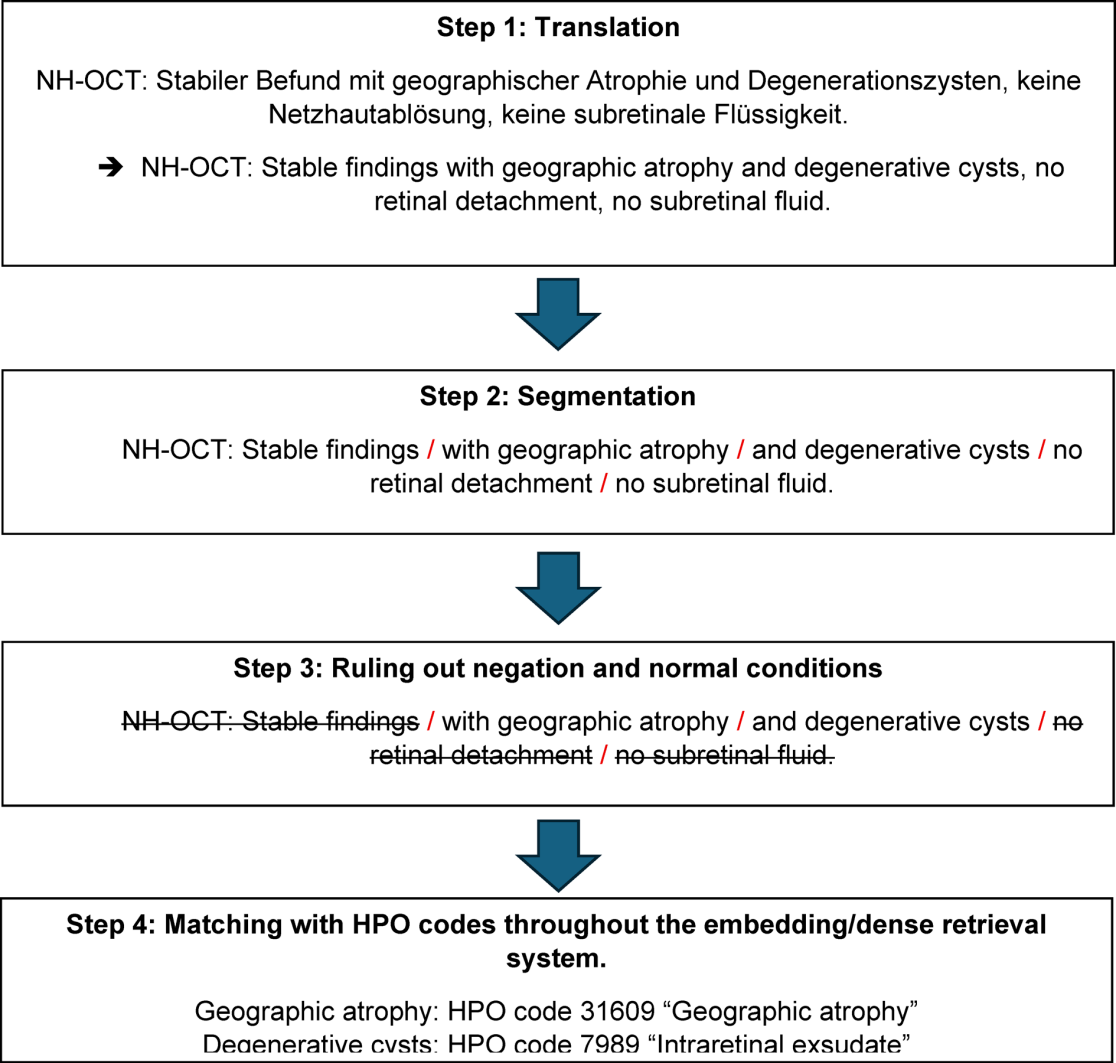
Workflow of the AI pipeline. HPO, human phenotype ontology; AI, artificial intelligence.

The AI pipeline was designed according to the following procedural framework (see Fig. 1):

*Step 1*—Data extraction and translation: Anonymized ophthalmological records were extracted from the administrative database of the Eye Center, Medical Center – University of Freiburg. A specific prompt was developed to translate free-text clinical notes from German to English to facilitate downstream processing, using in context-learning with hand-crafted few-shot examples. This was done by means of iteratively improving dedicated prompts for the anterior and posterior eye segments (see Supplementary Prompt S1 in Supplementary Information for the final prompt).

*Step 2*—Segmentation: Unstructured text describing the anterior and posterior eye-segments was algorithmically segmented into discrete sections, each corresponding to distinct phenotypic observations. This was achieved by iteratively refining dedicated LLM prompts for the anterior and posterior eye segments, primarily by adding few-shot examples (see Supplementary Prompt S2 in Supplementary Information for the final prompt).

*Step 3*—Exclusion and negation: The system screened segments to identify and exclude descriptions of normal findings. Furthermore, negation detection was applied to ensure that ruled-out pathologies were not incorrectly flagged as present. Again, the LLM-prompts were iteratively improved during training (see Supplementary Prompt S3 in Supplementary Information for the final prompt).

*Step 4*—Semantic retrieval and HPO mapping: Pathological findings were mapped to HPO codes using a dense retrieval system. This approach utilizes semantic embedding to match clinical descriptions with ontology terms even when terminology differs (e.g., mapping both “Amotio retinae” and “retinal detachment” to HP: 0000541). For the semantic matching of extracted findings to the HPO catalog, we employed a nearest neighbor search within the embedding space, specifically retrieving the single closest match (K = 1) for each segmented finding. Consequently, while each individual textual segment was mapped to exactly one HPO term, a single medical record could yield multiple HPO terms based on the number of distinct segments generated in Step 2. For the statistical evaluation, metrics including precision, recall, and F1-score were calculated strictly on the level of individual HPO terms across the entire dataset, rather than at the overarching case level.

### Construction of an augmented HPO catalog

To optimize the pipeline for ophthalmologic specificity, we continuously expanded the local HPO synonym catalog at the Eye Center, Medical Center—University of Freiburg. Whenever dense retrieval returned an incorrect HPO code, the corresponding term was added as a synonym to the correct HPO code. This error-correction procedure was applied during manual review of all terms extracted from approximately 10,000 medical reports. Through this process, the AI pipeline underwent iterative refinement using this dataset. Crucially, the reports used for training were distinct from those set aside for the validation study to prevent data leakage. It is important to notice that we did not train AI-models in the sense of fine-tuning but only iteratively improved in-context-learning and synonym-catalogues by means of manually reviewing all classification errors using an expert-in-the-loop approach.

Phenotypes identified during this review that were absent from the standard ontology were submitted to the original HPO consortium to supplement the global standard via GitHub. We curated distinct synonym catalogues the anterior and posterior segments separately (see Table 1).

**Table 1:** An example of the local HPO codes analog at The Eye Center at Medical Center, University of Freiburg. The single “0” indicated that the respective segment had been ignored. HPO, human phenotype ontology.

Anterior segment examples		Posterior segment examples	
HPO code	Annotation/synonym	HPO code	Annotation/synonym
1128	Hair on the graft	11504	Target maculopathy
45025	Lid fissure width: 10 mm, 11 mm	31151	Tractional intraretinal cysts on macular OCT
11457	Eyelash loss	11530	Adhering flap
6000709	Absent Bell phenomenon	25314	Choroidal nevus at the inferior vascular arcade
500081	Central capsulorhexis	11530	Bridge vessel with elevated margins
525	Temporal punctate iris defect	200071	Areas of degeneration at 7 o’clock
200020	Three linear scratches from 6 o’clock to 9 o’clock, mid-peripheral and peripheral	7777	Double row laser burns
0	Sclera is compressible	30499	Drusen within atrophic areas
9743	Double eyelashes	40049	Fluid at the temporal edge of the atrophic zone
100019	Inferior cataract with spicule	7777	Inferior temporal laser grid from 6:30 to 9 o’clock
100018	Red cataract	8028	Macular cystic lesions
12905	Wide-set eyes	1177	Peripheral retinal resection
500074	Elevation deficit	31805	Roth spot
31705	Forced head position	500061	Multiple avascular areas in zone III
7648	Lens with diffuse, snow-like opacities	1777	Fresh laser burns
0	Motility	11530	Floating retinal flap
11488	Pigment on endothelium (left > right)	31805	Streak-like bleeding at the inferior margin
40004	Substance defect with pooling	0	Visualisation improved
2664	Tumor with increased vascularity	0	Visualisation significantly reduced
7734	Suspected dacryoadenitis	30609	Fibrosis in the outer neurosensory retina

### Validation study

To evaluate the performance of the pipeline, a validation set comprising 175 anonymized medical records was selected entirely at random using PostgreSQL database row shuffling. This ensured an unbiased, real-world distribution of clinical texts, independent of the frequency of specific HPO codes. These records were processed by the AI pipeline to generate automated HPO annotations. Concurrently, the same records underwent manual review by an experienced ophthalmologist to establish a ground truth dataset.

Statistical performance was assessed by comparing the AI-generated annotations against the manual ground truth. The following metrics were calculated:

Descriptive statistics: Average number of HPO terms per record.

Jaccard similarity: To measure the overlap between the predicted and actual term sets.

Precision, recall, and F1-Score: To evaluate the accuracy and sensitivity of the extraction.

All statistical analyses were conducted using R software (R Foundation for Statistical Computing, Vienna, Austria).

### Ethics and data protection

All patient data utilized in this study were fully anonymized prior to processing. Due to the retrospective nature of the study and the use of anonymized data, the requirement for individual informed consent was waived. All procedures were conducted in accordance with relevant guidelines and regulations. The study protocol was reviewed and approved by the Ethics Committee of the Albert-Ludwigs-Universität Freiburg (Application No. EK-Freiburg: 25-1289-S1-retro).

## Results

The final architectural details for the AI pipeline are shown in Supplementary Information. Supplementary Prompt S1 (Translation), Supplementary Prompt S2 (Segmentation) and Supplementary Prompt S3 (Negation and Extraction) depict the final LLM prompts at the end of the iterative training period.

The validation resulted in an average of 2.53 HPO terms in the manual annotation (ground truth) and 2.52 HPO terms in the annotation generated by the AI pipeline. In total, 342 terms were identified in the ground truth and 341 terms by the AI pipeline. The agreement between manual and automated annotation with the AI pipeline was evaluated with following results: the median Jaccard similarity was 0.63, the median precision 0.80, the median recall 0.80, and the median F1-score 0.77. The results are demonstrated in Table 2.

**Table 2:** Results of the validation study (*n* = 175). HPO, human phenotype ontology; AI, artificial intelligence.

	AI pipeline	Manual annotation
HPO terms per medical record (mean)	2.52	2.53
Identified HPO terms in total	341	342
	Jaccard-Similarity: Median 0.67	
	Precision: Median 0.83	
	Recall: Median 0.82	
	F1-Score: Median 0.80	

### Error analysis of misclassifications

An analysis of notable misclassifications revealed that errors were predominantly caused by the absence of highly specific ophthalmologic terms in the standard HPO catalog at the time of the study. For instance, clinical concepts such as ‘Salzmann’s nodular degeneration’ were not yet available in the ontology. Because our retrieval system was constrained to a nearest neighbor search (K = 1), these absent concepts were unavoidably assigned to the closest existing neighbor in the semantic embedding space, leading to an imprecise or related parent-term match. All such missing clinical phenotypes identified during this study have been submitted to the original HPO consortium via GitHub for inclusion in upcoming releases.

## Discussion

The findings of this study demonstrate that our locally deployed AI pipeline is both effective in extracting ophthalmic phenotypes. A critical architectural advantage of this system is the implementation of a dense retrieval framework (embedding-based search) combined with a small language model, rather than relying exclusively on generative LLMs for extraction.

While generative models offer flexibility, they are prone to “hallucinations”—the generation of plausible but factually incorrect or non-existent medical terms. By utilizing dense retrieval, we constrain the system to map clinical text exclusively to a curated, fixed catalog of verified HPO codes. This approach effectively “grounds” the AI, ensuring that every output corresponds to a legitimate ontological entity. This control mechanism is particularly vital in medical contexts where precision is paramount. The success of this strategy is reflected in our performance metrics: a precision of 0.83 indicates a minimal rate of false positives, suggesting that the system successfully avoided hallucinating incorrect phenotypes while maintaining high accuracy.

In benchmarking against expert manual annotation (ground truth), the pipeline achieved robust results, with a median Jaccard similarity of 0.67, recall of 0.82, and F1-score of 0.80. A Jaccard similarity value of 0.67 signifies a substantial level of overlap between manual and AI-derived annotations. In complex biomedical NLP tasks, perfect overlap is rarely achievable due to semantic nuances and inter-annotator variability; however, values exceeding 0.60 are generally regarded as indicative of strong agreement^13^. Furthermore, the F1-score of 0.80 aligns with state-of-the-art benchmarks for automated phenotype annotation tools^14^. These findings provide strong evidence that the pipeline is reliable for extracting phenotypic data from unstructured ophthalmological records.

A significant advantage of the proposed AI pipeline is its potential to facilitate GDPR compliance through local, on-site data processing. One of the primary obstacles to multi-center research and the dissemination of medical registries is the risk of exposing protected health information.

By converting narrative clinical notes into standardized HPO codes, the pipeline effectively de-identifies the documentation at the source. Because the system operates on local infrastructure, sensitive patient data never leaves the hospital’s secure environment. As AI-capable hardware becomes increasingly powerful and cost-effective, even smaller ophthalmology practices will soon be able to deploy these systems on-site, ensuring high-level data protection without sacrificing the ability to contribute to large-scale research registries.

A remaining challenge is the linguistic variability inherent in clinical documentation across different institutions. Medical terminology, abbreviations, and documenting styles can vary significantly between hospitals. A system optimized for the “local dialect” of one university hospital may encounter domain shifts when applied elsewhere.

However, this limitation is manageable within our modular framework without deep technical knowledge as the prompts can be adopted in natural language by domain experts themselves to accommodate regional terminologies without altering the core architecture. The dense retrieval system allows for rapid adaptation; the retrieval catalog can also be expanded with local synonyms. This adaptability ensures that the pipeline can be scaled across different clinical sites with a brief “calibration” phase.

HPO offers hierarchical, expandable term sets for phenotypic features that can be directly mapped to clinical presentations, thereby complementing the diagnostic breadth of another medical coding system such as the International Classification of Diseases, 10th Revision (ICD-10 codes). Recent research by Böhringer et al. demonstrated that the automated inference of ICD-10 codes from German ophthalmologic physicians’ letters using natural language processing (NLP) achieves good performance^15^. However, it faces intrinsic limitations in granularity and semantic capture compared to ontology-based approaches like those utilizing human phenotype ontology (HPO). These findings underscore the necessity for complementary systems that span the domains of diagnostic classification and deep phenotyping, thereby ensuring comprehensive informatics support within the realm of ophthalmology.

A limitation of the current study is the lack of formal validation metrics for the individual pre-processing stages of the AI pipeline (translation, segmentation, and negation detection). Because our evaluation focused on end-to-end performance by comparing the final HPO output against expert ground truth, we cannot definitively quantify the isolated error rates of Steps 1 through 3. Future studies should aim to evaluate these steps independently to identify specific computational bottlenecks. Furthermore, regarding the implications of incorrect HPO assignments, it is important to note that systematic classification errors by the AI pipeline will generally not confound longitudinal or cross-sectional studies, provided the bias remains consistent across time and comparison groups. However, such systematic misclassifications would negatively impact the validity of prevalence studies, where absolute diagnostic accuracy and exact phenotypic counts are strictly required.

In summary, this study demonstrates the feasibility and accuracy of automatically extracting ophthalmologic phenotypes from routine medical records using a small language model. The system’s dense retrieval architecture serves as a crucial safeguard against AI hallucinations by grounding outputs in a validated ontology.

Looking forward, the rapid evolution of generative AI and hardware acceleration promises to further enhance this pipeline. The recent availability of advanced reasoning models, such as the OpenAI oss-series, alongside the proliferation of high-performance, AI-optimized local hardware, will significantly improve the pre-processing stages of our pipeline—specifically segmentation and negation detection. As these language models become more capable of handling complex nuances, the robustness of automated phenotyping will continue to grow.

## Supplementary Information

Below is the link to the electronic supplementary material.

Supplementary Material 1

## Data Availability

The data from this manuscript are not publicly available due to privacy concerns expressed by the healthcare providers. Despite anonymization, individual access to the data can be arranged if appropriate precautionary measures are taken, ensuring compliance with the German data protection laws. Access will be granted only under strict conditions to safeguard patient privacy and data security. Please send requests via email to daniel.boehringer@uniklinik-freiburg.de.
