# Supplementary material for: Automated phenotyping of ophthalmologic diseases from routine medical records using small language models and the human phenotype ontology (HPO)
Source: Sci Rep. 2026 May 9;16:14682. doi: 10.1038/s41598-026-51512-z (PMC13157479; doi:10.1038/s41598-026-51512-z)
Supplement: Supplementary file 1 — Supplementary Material 1 [file 41598_2026_51512_MOESM1_ESM.docx]

**Supplementary Information**

**Supplementary Prompt S1: Final prompt for English translation (an example for posterior segment)**

You are a brilliant ophthalmologist. Your job is to translate a German medical term (taken from the eyelid, conjunctiva / sclera, cornea, anterior segment, iris, pupil and lens section of a medical report) to professional medical English. Coin the term so it could be in the Human phenotype ontology.

Answer "unknown" if the term does not have enough context to be a medical term.

Use these hints for your translation:

<hints>

Faden -> suture

Einblick -> visualisation of intraocular structures

Kernsklerose -> Nuclear sclerosis of the lens

Nachstar ->seondary cataract

gestippt -> punctate lesions

Stippung -> Stippling

...donesis -> loosened

fragl. -> questionable

Sickerkissen -> filtering bleb

Guttae -> Guttae

Rubeosis -> Iris neovasculariation

Korectopie -> corectopia

Glaskörperschlieren -> Vitreous condesations

Hornhaut offen -> corneal erosion

Seidel -> Seidel

cornea guttata -> cornea guttata

hyposphagma -> hyposphagma

Oxford grade I -> Oxford grade I

Fleischerring -> Fleischer ring

Kayser-Fleischer-Kornealring -> Fleischer ring

V.a. -> suspected

Kaurnkel -> caruncle

Transplantatdehiszenz -> graft dehiscence

Pupillenentrundung -> pupillary irregularity

Haut verhärtet -> skin indurated

Eisenlinie -> iron line

Hornhaut aufgesteilt -> corneal steepening

Bindehaut mit Nävus -> conjunctival nevus

Endothelbeschläge -> keratic precipitates

Endothelpräzipitate -> keratic precipitates

Pupille verzogen -> pupil distorted

Gerstenkorn -> hordeolum

Chalazion -> chalazion

Altersentsprechend -> consistent with age

Corneal pooling -> Dellen

Bindehaut unterblutet -> subconjunctival hemorrhage

Pupille spielt -> Pupil reacts

Vorderkammer reizarm -> Anterior chamber unreactive

KL-Sitz -> placement of the contact lens

Ptosis -> ptosis

Epithel zu -> Epithelium closed

Blutbeschlag -> blood staining

Festoons -> festoons

BUT 3s -> break up time 3 seconds

verbandslinse -> bandage lens

hintere schalentrübung -> posterior cataract

RAPD -> Relative Afferent Pupillary Defect

Stauchungsfalten -> compression folds

Epiphora -> epiphora

lidkante verdickt -> eyelid margin thickended

Lidschlag -> blinking

verbreitertes Randschlingennetz -> widened vascular network at the limbus

Hornhaut versalbt -> cornea greasy

Iridodonesis -> iridodonesis

Vogt-Linien -> Vogt-striae

Bulbus tonisiert -> eyeball toned

blande -> mild

Minimonoka -> Minimonoka

Lichtweg -> anterior chamber flare

Tx in loco -> graft in place

Lidschluss -> eyelid closure

Pigmentepithelversprengung -> RPE dispersion

Speichentrübung bis ans Zentrum -> spoke-like opacities to the center

Eversio puncti lacrimalis -> Eversion of the lacrimal punctum

Rindentrübung -> cortical opacities

Blut am Endothel -> Blood at corneal endothelium

teigige Schwellung -> doughy swelling

Glissening -> Glissening

Krokodilchagrin -> crocodile chagrin

Iristransillumination -> iris transillumination

HST -> posterior lens opacification

PUK -> peripheral ulcerative keratitis

TWS -> lacrimal drainage system

BH -> conjunctiva

HH -> cornea

LIPCOF->conjunctival folds

UTP -> lower lacrimal punctum

VK -> anterior chamber

PEX -> pseudoexfoliation

BA -> both eyes

RA -> right eye

LA -> left eye

s.c. -> subconjunctival

DD -> Differential diagnosis

EKN -> corneal suture

subtarsal regelrecht -> subtarsal area normal

Wimpernrarefizierung -> eyelash thinning

Lider regelrecht -> Eye lids normal

hard stop -> hard stop

Epigard-Reste einliegend -> Epigard remnants in place

Lidkanteneinziehung -> notching of the lid margin

Narbenektropium -> cicatricial ectropion

Starkes Kneifen -> Blepharospasm

Tränenfilm: BUT beidseits 0 s -> tear break up time 0 seconds in both eyes

In Miosis: Zirkulär Randunschärfe -> In miosis: circular blurring of the pupillary margin

Benetzungsstörung -> tear film instability

Khodadoust-Linie nasal -> Khodadoust line nasally

etwas Pigment am Endothel -> some pigment on the endothelium

Kirchenfenster-Phänomen -> church window phenomenon

Hypotropie -> hypotropia

Railroad tracks -> Railroad tracks

in loco -> in place

Lidhämatom -> eyelid hematoma

beim Ektropionieren Follikel -> follicles visible on eversion of the eyelid

Resterosio -> residual erosion

Plica schwellung -> swelling of the plica semilunaris

Linse mit Speichen -> lens with spoke like opacities

AWDP in Miosis -> on patient's wish examination only in miosis

Tunnel dicht -> Incision is self-sealing

Moll Zyste -> cyst of moll

Exotropie -> Exotropia

Esotropie -> Esotropia

</hints>

Using these hints, now translate this term:

"_INPUT_".

Just give the english translation, nothing else, no notes, just the translation, please!

**Supplementary Prompt S2: Final prompt for segmentation**

your are a brilliant ophthalmologist. your job is to split this text taken from a medical report into separate findings. E.g. this

<example>"Lider reizfrei, kein Druckschmerz, Bindehaut reizfrei, Hornhaut mit Trübungen, die bis ins anteriore Stroma reichen, deutliche Stippung v.a. im Zentrum. Sensibilität vollständig aufgehoben, keine Erosio, versalbt, BUT 0 sec, PEX, Vorderkammer steht Hyphäma 0,5mm, IE offen, Linse getrübt, Kernsklerose, Speichen" should become:

"Lider reizfrei|kein Druckschmerz|Bindehaut reizfrei|Hornhaut mit Trübungen, die bis ins anteriore Stroma reichen|Hornhaut mit deutlicher Stippung v.a. im Zentrum|Hornhautsensibilität vollständig aufgehoben|Hornhaut ohne Erosio|Hornhaut versalbt|Tränenfilm: BUT 0 sec|Pupille mit PEX|Vorderkammer steht|Vorderkammer mit Hyphäma 0,5mm|Iridektomie offen|Linse getrübt|Linse getrübt|Linse mit Kernsklerose|Linse mit Speichen"

</example>.

As you see in <example> it is important to preserve context when feasible upon splitting (what anatomical structure ist refered to).

Now segment this sentence accordingly, separated by "|":

"_INPUT_".

nothing else, just the segmentation please!

**Supplementary Prompt S3: Final prompt for negation and extraction**

your are a brilliant ophthalmologist. your job is to remove all negated terms and terms from <list> that describe the absence of something. give the list back unmodified if there is nothing to remove.

<list>

_INPUT_

</list>

please give just the cleaned list with items separated by '|', no notes, no explanation!
